# Setting the standard: multidisciplinary hallmarks for structural, equitable and tracked antibiotic policy

**DOI:** 10.1136/bmjgh-2020-003091

**Published:** 2020-09-23

**Authors:** Claas Kirchhelle, Paul Atkinson, Alex Broom, Komatra Chuengsatiansup, Jorge Pinto Ferreira, Nicolas Fortané, Isabel Frost, Christoph Gradmann, Stephen Hinchliffe, Steven J Hoffman, Javier Lezaun, Susan Nayiga, Kevin Outterson, Scott H Podolsky, Stephanie Raymond, Adam P Roberts, Andrew C Singer, Anthony D So, Luechai Sringernyuang, Elizabeth Tayler, Susan Rogers Van Katwyk, Clare I R Chandler

**Affiliations:** 1 School of History, University College Dublin, Dublin, Ireland; 2 Oxford Martin School, University of Oxford, Oxford, Oxfordshire, UK; 3 Department of Public Health and Policy/ Institute of Infection and Global Health, University of Liverpool, Liverpool, Merseyside, UK; 4 School of Social and Political Sciences, Faculty of Arts and Social Sciences, The University of Sydney, Sydney, New South Wales, Australia; 5 Princess Maha Chakri Sirindhorn Anthropology Center, Bangkok, Thailand; 6 Antimicrobial Resistance and Veterinary Products Department, World Organisation for Animal Health, Paris, Île-de-France, France; 7 Irisso, Paris-Dauphine University, PSL, INRAE, Paris, Île-de-France, France; 8 Center for Disease Dynamics Economics and Policy, Washington, DC, USA; 9 Department of Infectious Disease, Imperial College London, London, UK; 10 Institute for Health and Society, Dept. of Community Medicine and Global Health, University of Oslo, Oslo, Norway; 11 Geography, College of Life and Environmental Sciences and Wellcome Centre for Cultures and Environments of Health, University of Exeter, Exeter, Devon, UK; 12 Global Strategy Lab, Dahdaleh Institute for Global Health Research, Faculty of Health and Osgoode Hall Law School, York University, Toronto, Ontario, Canada; 13 Institute for Science, Innovation and Society, School of Anthropology and Museum Ethnography, University of Oxford, Oxford, Oxfordshire, UK; 14 Infectious Diseases Research Collaboration, Kampala, Central Region, Uganda; 15 School of Law, Social Innovation on Drug Program, Boston University, Boston, Massachusetts, USA; 16 Department of Global Health and Social Medicine, Harvard Medical School, Boston, Massachusetts, USA; 17 Department of Tropical Disease Biology, Liverpool School of Tropical Medicine, Liverpool, Liverpool, UK; 18 Pollution, UK Centre for Ecology & Hydrology, Wallingford, UK; 19 Department of International Health, Johns Hopkins University Bloomberg School of Public Health, Baltimore, Maryland, USA; 20 Innovation + Design Enabling Access (IDEA) Initiative, ReAct - Action on Antibiotic Resistance, Baltimore, Maryland, USA; 21 Faculty of Social Sciences, Mahidol University, Salaya, Nakhon Pathom, Thailand; 22 Global Coordination and Partnerships, AMR Division, World Health Organisation, Geneva, Switzerland; 23 Global Strategy Lab, York University, Toronto, Ontario, Canada; 24 Department of Global Health and Development, London School of Hygiene & Tropical Medicine, London, UK

**Keywords:** health policies and all other topics, infections, diseases, disorders, injuries, public health

## Abstract

There is increasing concern globally about the enormity of the threats posed by antimicrobial resistance (AMR) to human, animal, plant and environmental health. A proliferation of international, national and institutional reports on the problems posed by AMR and the need for antibiotic stewardship have galvanised attention on the global stage. However, the AMR community increasingly laments a lack of action, often identified as an ‘implementation gap’. At a policy level, the design of internationally salient solutions that are able to address AMR’s interconnected biological and social (historical, political, economic and cultural) dimensions is not straightforward. This multidisciplinary paper responds by asking two basic questions: (A) Is a universal approach to AMR policy and antibiotic stewardship possible? (B) If yes, what hallmarks characterise ‘good’ antibiotic policy? Our multistage analysis revealed four central challenges facing current international antibiotic policy: metrics, prioritisation, implementation and inequality. In response to this diagnosis, we propose three hallmarks that can support robust international antibiotic policy. Emerging hallmarks for good antibiotic policies are: Structural, Equitable and Tracked. We describe these hallmarks and propose their consideration should aid the design and evaluation of international antibiotic policies with maximal benefit at both local and international scales.

**Summary box**

- The global crisis of antimicrobial resistance has led to a proliferation of expert reports and national and international antibiotic action plans.
- Implementing international antibiotic policy that is meaningful in different social, cultural and economic contexts continues to prove challenging.
- Our multidisciplinary analysis has identified four critical challenges of metrics, prioritisation, implementation and inequality for international antibiotic policy-making.
- We propose a corresponding SET of basic hallmarks of good antibiotic policy, which we define as Structural, Equitable and Tracked.
- Our SET of hallmarks can orientate disciplinary debates and provide a framework for developing robust international interventions.

## Introduction: antimicrobial resistance as a biosocial problem

Since their introduction in the 1930s, antibiotics have acquired infrastructural importance in global health and food production.^1^ However, antibiotic reliance comes with a trade-off: using antibiotics accelerates the emergence of antimicrobial resistance (AMR), which diminishes their future effectiveness. This makes effective antibiotics a precious ‘global common-pool resource’,^2–6^ which can benefit humanity but will provide diminishing benefits if we fail to coordinate plans for preservation (a tragedy of the commons).

Over the past decade, rising concern about AMR has highlighted the need for collective action to protect our antibiotic ‘commons’.^2 7–12^ Governmental and non-governmental organisations have devoted substantial resources to tackling AMR and preserving antibiotic effectiveness on national and international stages.^13^ Increasing attention and funding have been accompanied by a proliferation of expert reports and policy proposals.^14^ While these AMR-focused initiatives have succeeded in achieving a clear international consensus on the need for action, many lament a lack of action in practice, dubbed an ‘implementation gap’. Substantial uncertainties also remain about the effects of different policy interventions, how international policies could be enforced globally, and who will pay in the long term. The main difficulty with managing the antibiotic commons seems to lie in jointly addressing the complex biological and social (understood here to be historical, political, economic and cultural) dimensions of AMR. Policy formation for the latter dimension is particularly challenging because it entails addressing different metrics, meanings and challenges in different settings.

We propose a new wide-angle approach to AMR-focused antibiotic regulation. Two basic questions guide our reflective process: (A) Is a universal approach to AMR policy and antibiotic stewardship possible? (B) If yes, what hallmarks characterise ‘good’ antibiotic policy?

To answer these questions, we have developed an innovative heuristic evaluation framework, which accounts both for AMR’s biological and social facets. Our approach was informed by the numerous national and international reports proposing various principles for antibiotic policy-making^2 10 11 15–17^ and the historical success of the 3Rs (Reduction, Refinement and Replacement) that restructured laboratory animal testing protocols. Developed as principles of ‘good animal experimentation’ in 1959, the 3Rs were concrete and aspirational enough to trigger a progressive evolution of protocols and dialogue in a contested policy arena.^18^(So *et al* have proposed three Rs for antimicrobial development).^19^

Applying this approach to AMR and antibiotic policy, our collaborative multidisciplinary analysis employed a three-stage evidence gathering, evaluation and consultation process (box 1), which consisted of: (1) asking selected medical humanities and sciences researchers whether they believe antibiotic policies could feasibly be guided by a universal set of guiding principles, and what they consider those guiding principles could be; (2) identifying possible hallmarks of good policy with a broad group of stakeholders from academia, medicine, animal/plant production, policy and funding bodies at an international workshop and (3) refining identified hallmarks in light of multidisciplinary feedback.

Box 1 **Three stage evidence gathering, evaluation and consultation process**

**Stage 1:**

Following a 2018 Social Science and antimicrobial resistance (AMR) Research Symposium at the British Academy in London,^162^ a correspondence group of fourteen experts from the humanities, social, environmental and medical sciences reflected on whether there could be universal principles of international antibiotic policy making and hallmarks of ‘good’ policy.

**Stage 2:**

Identified policy challenges and core hallmarks of successful intervention informed a preliminary paper, which was circulated among correspondents and attendees of a multidisciplinary 2-day workshop in London in March 2019.

During presentations, small group breakout sessions, and group discussions, participants reflected on the preliminary paper’s four identified problem areas for antibiotic regulation: (A) metrics (defining and measuring AMR, antibiotic usage and performance indicators for interventions); (B) prioritisation (prioritising specific forms of antibiotic use over others); (C) implementation (developing and implementing interventions that are meaningful in high-income, medium-income and low-income settings); (D) inequality (formulating interventions that take into account global disparities of wealth, infectious disease and AMR burdens and access to antibiotics as well as effective health, water, sanitation and hygiene systems).

There was agreement on these interlinked problems but the four provisional hallmarks proved more contentious: (1) antibiotic policymaking should take into account antibiotics’ infrastructural relevance in medicine and food production; (2) should increase the microbial *resilience* of health and food systems to diminish the need for antibiotics; (3) be responsive to evolving knowledge regarding AMR and (4) relational in its acknowledgement of differing local challenges and capacities.

Participants agreed that there was no single solution to AMR but felt that hallmarks needed to be integrated and go beyond preserving the *status quo*. Key to more effective policymaking has to be an acknowledgement of antibiotics’ primary utility for global health and food production, which consists in their ability to reduce mortality and morbidity resulting from treatable infections. To preserve this utility, the overriding aim of any antibiotic policy must therefore be (1) to maximise and maintain access to effective treatments for infections, which includes the development of new treatments—while (2) reducing the need for antibiotic use by preventing infections, reducing antibiotic dependencies in healthcare and food systems, and minimising the environmental load of antibiotics and resistant bacteria.

**Stage 3:**

A refined version of new Structural, Equitable, Tracked Hallmarks was circulated among correspondents and participants, who were invited to be coauthors on the paper.

We believe that the resulting heuristic compass based first on the identification of central challenges of antibiotic policy-making (metrics, prioritisation, implementation, inequality) and second on the formulation of a corresponding Structural, Equitable and Tracked (SET) of hallmarks of good antibiotic policy, which we define as *SET* can orientate disciplinary debates and provide a framework for robust international interventions.^10 20 21^

## The four central challenges of antibiotic regulation

### Metrics

Despite long-standing regulation attempts, there remains substantial uncertainty about basic metrics surrounding antibiotic usage and AMR as well as about how to correlate measurements in a way that can inform meaningful policy formulation at the national and international levels.^22^

#### How to define resistance?

Since the first warnings about ‘drug fastness’ in microorganisms in 1907,^23 24^ there has been no clear transdisciplinary or international consensus on how to define AMR. The constantly evolving nature of AMR, the introduction of new drugs and the different availability and use of antibiotics means that terms like drug sensitive, intermediate resistant or resistant, mean different things in different regional contexts. There is also no transdisciplinary consensus on whether to define AMR according to predefined clinical breakpoints, minimum inhibitory concentrations, epidemiological cut-offs, pharmacokinectic/pharmacodynamic models, the presence or absence of resistance-conferring genetic elements or clinical impact on patients or animals. Disagreement over this latter point is highlighted by the common absence of drug-resistant infection as an official cause of death. Historically, the lack of consensus over AMR has led to differing microbiological and public health definitions of AMR with the former measuring incremental changes of microbial susceptibility and the latter measuring instances of treatment failure at predefined dosages.^25–27^

#### Whose methods count?

Internationally, the establishment of WHONET (est. 1989) and WHO’s Global Antimicrobial Resistance Surveillance System (GLASS, est. 2015) marked important efforts to standardise AMR reporting in humans and make data comparable.^28 29^ However, coordination problems remain: testing protocols by influential bodies like the European Committee on Antimicrobial Susceptibility Testing (EUCAST) and the US-based Clinical Laboratory Standards Institute (CLSI) occasionally diverge. Resulting international monitoring differences are exacerbated by issues of access (see problem area Inequality).^30^ EUCAST guidelines and updates are available free of charge. By contrast, CLSI guidelines and updates are often pay for use, which makes it difficult for resource poor laboratories to keep protocols up to date and feedback local AMR data into international databases.^31^ In low and middle income countries (LMICs), surveillance is often further complicated by lack of access to laboratory equipment, service contracts and paywalled literature.^32^ Ensuing disparities in global AMR reporting mean that international reports disproportionately reflect data from resource-rich settings and a limited number of well-studied low-income sentinel sites with international healthcare infrastructure investment.^33^ Resulting international stewardship and policy guidelines may, however, be of limited use in understudied resource-poor settings with different AMR ecologies and no access to key antibiotics.

#### What is relevant antibiotic use data for AMR?

Capturing relevant data on antibiotic use to inform AMR efforts has its own challenges. Starting in the 1990s, various high income countries (HICs) began to compile antibiotic usage data.^34–37^ However, data collection methods continue to vary. In the case of antibiotic usage in animals, the World Organisation of Animal Health (OIE) has begun to collect data on antibiotic sales intended to be used in animals and usage data since 2015. However, despite ongoing progress, data gaps remain with almost 25% of 182 OIE Member Countries not reporting quantitative and most reporting antibiotic sales and imports data but no data on use for the fourth OIE round of data collection.^38^ Reporting differences are also common among HICs. The EU developed a standardised metric to correlate antibiotic sales with the volume of animal production (mg/population correction unit) from 2010 onwards.^39^ Individual EU countries like Britain and Denmark publish not just sales but farm usage and prescription data for certain livestock categories.^40 41^ By contrast, the US Food and Drug Administration (FDA) publishes sales data only in broad categories of drug class by species but no usage data, which complicates AMR risk assessment.^42^

Correlating usage and AMR data is even more challenging. In its 2018–2019 report, the English Surveillance Programme for Antimicrobial Utilisation and Resistance (ESPAUR) showed a significant reduction in antibiotic prescriptions but an increase in antimicrobial-resistant infections for the seven priority bacterial pathogens reported.^43^ In 2017 and 2019, longitudinal studies of bloodstream infections in Malawi showed a long-term reduction of overall infections but a rise of antibiotic resistance in remaining infections.^33 44^ To be useful, aggregated metrics of antibiotic usage (including class of antibiotic) across infection types need to be contextualised with outcome and population health metrics, such as infection, resistance and morbidity/mortality rates.^45–47^

Understanding what the implications of specific forms of antibiotic usage (eg, prophylaxis, therapy, growth promotion) in different environments are for AMR and health outcomes is similarly crucial for policy formulation. Rising numbers of point prevalence studies and whole-genome sequencing are enhancing our knowledge of drivers and variations of AMR over time and in different areas across the world.^48–51^ However, our wider understanding of the evolutionary factors underlying AMR levels is still fragmentary as is our understanding of which stewardship interventions might make how much of a difference: in some cases like targeted 1950s antibiotic prescription bans at St Bartholomew’s Hospital in London^52^ or Denmark’s 1990s ban of lower-dosed avoparcin and tylosin animal growth promoters, reducing selection pressure by a specific type of drug usage led to a marked reduction, but not a complete disappearance, of correlating AMR.^53 54^ In other cases, antibiotic reductions may take years to manifest in terms of reduced AMR—as highlighted by the UK’s 2019 ESPAUR report and experiences in Scandinavian countries.^43 53 55^ Finally, some usage reductions may come too late to shift the evolutionary balance back in favour of microbial sensitivity as occurred with China banning the use of colistin growth promoters in 2016^56^ following a report of the transferable mobilisable colistin resistance *MCR-1* gene.^57^ Subsequent reports, however, showed that the gene was detectable in strain collections from more than 30 countries and was already circulating in *Escherichia coli* in China in the mid 1980s.^58^ The lack of uniformly comparable data makes it more difficult to evaluate trade-offs in various policy options.

#### How to develop meaningful key performance indicators?

While uncertainty remains about the degree to which interventions will be effective, reducing the overall amount of antibiotics used in health and animal and plant production systems is a key component of most AMR action plans.^11 13 59^ Over the past two decades, international bodies have attempted to decide which antibiotics to protect from overuse. Since 2004, WHO, OIE and the Food and Agriculture Organization (FAO) have begun using the categories of critically important antimicrobials (CIAs), highly important antimicrobials and important antimicrobials—although differences in the categorisation of the same drugs for veterinary and medical usages can remain.^60 61^ In 2019, the WHO’s Essential Medicines List added the AWaRe categorisation, to meet a need to recognise those medicines that should be *Accessed* as first-line narrow-spectrum treatment for particular conditions, and those with higher risks of becoming resistant that should be used Watchfully and those that should be Reserved for last-line treatment.^62^ This framework can be used as an index—the ratio of Access to Watch and Reserve medicines—to compare prescribing practices in different contexts, which goes some way to balancing the different challenges of improving access and restricting excess when setting targets.^63^ However, measurement of antibiotic usage remains a challenge. Although WHO and OIE have standardised methodologies to collect country-level antimicrobial usage in humans and animals for global reporting and consulting companies like IQVIA gather and sell additional proprietary data,^64 65^ more granular level detail about antibiotic usage on farms and in particular clinical and residential settings is required for targeted reduction strategies.^66 67^

Alongside improved usage data, deciding which microbes, resistance genes and AMR reservoirs to monitor in medical, animal and plant production, and environmental settings is similarly important for the formulation of meaningful policy interventions. For humans, WHO published a list of priority pathogens to monitor and target with antibiotic development efforts in 2017.^68^ For animals, the OIE Member Countries agreed on harmonised lists for both terrestrial and aquatic animals.^69 70^ However, it remains difficult to prioritise which sentinel organisms to survey and where potentially important AMR reservoirs are located due to different health threats in HICs, MICs and LICs, lacking clinical surveillance infrastructure in many LMICs and of environmental surveillance in the most countries, regionally skewed reporting that often centres on urban clinical settings, and limited diagnostic capabilities for non-classic pathogens.^71 72^ Furthermore, particular microbes such as *Staphylococcus aureus* and *E. coli* may or may not be causing disease, and therefore, their measurement without corresponding disease burden data might be misleading.

Discussed in more detail below (see Tracked), one recently proposed solution for human health might be to integrate AMR and infection burden measurements by tracking two priority organisms in bloodstream infections as part of United Nations (UN) Sustainable Development Goal (SDG) 3.d.1. This new AMR-specific SDG could help build laboratory capacity in resource-poor settings and spur the development of further integrated AMR-specific metrics. Another approach might be to strengthen point prevalence studies for specific sentinel organisms in LMIC settings.^73^ (Consultations on sentinel organisms and sentinel sites are ongoing by GLASS and the point prevalence survey).

The described temporal and contextual challenges of defining resistance, measuring antibiotic usage in relation to AMR and concerning which microbes to focus on also raise important questions when it comes to defining benchmarks or key performance indicators (KPIs) for policy initiatives: how far should antibiotic sensitivity be restored or preserved for a measure to be considered successful? Should the performance of a policy be linked to its reduction of antibiotic usage or should the KPI be demonstrated impact on human health? Should success be defined as the stabilisation or decline of AMR in specific culturable pathogens? Given the mobility of resistance genes and the One Health dimensions, should a metric of success be the abundance or prevalence of a particular resistance gene in the wider environment, determined through quantitative PCR methods^74^? How much time should policies have to achieve their goals?

### Prioritisation

Overemphasis on surveillance data itself can serve to obscure different regional capabilities, underlying political interests and competing needs in animal and human medicine.^75^ In addition to defining meaningful metrics, a significant challenge facing international antibiotic regulation is which form of antibiotic use to prioritise in the face of time-limited microbial sensitivity to most drugs and heterogeneous epidemiological, social, economic and material contexts. The challenge of prioritisation comprises spatial and temporal components.

Spatial: since the 1940s, regulators have tried to protect important antibiotics by restricting their use,^76^ but this path-dependent prioritisation has been enacted differently across varying local, regional and national settings. Antibiotics’ infrastructural importance in global healthcare and food production means that a large number of sectors with different needs depend on routine antibiotic access.^1^ In the case of Europe and North America, historians have shown that distinct national antibiotic usage patterns have become socially entrenched over decades.^77–81^ To this day, antibiotic usage patterns vary across Europe and North America despite both regions' close economic, political and cultural ties.^82 83^ Differences of usage are even more substantial between HICs and LMICs with patients in the latter countries often depending to a much stronger degree on the efficacy of a limited number of locally available, affordable and easily administrable drugs—particularly in areas where there is no access to professional healthcare facilities.^84–86^ If different countries, sectors and even local healthcare facilities^87^ use different antibiotics for a variety of biological and social reasons, whose form of antibiotic use should receive priority?

The same problems of spatial prioritisation hold true in animal and plant production.^34 88^ One example of spatialised inequality of access are the polymyxin antibiotics (eg, colistin, discovered in 1949). After using the drugs to treat gram-negative infections in humans, HICs greatly reduced use of polymyxins in favour of less toxic carbapenems from 1980 onwards.^89^ Although limited use of ‘old’ colistin continued in HICs, their low cost and lacking HIC demand for human medicine led to aggressive pharmaceutical marketing and large-scale uptake for growth promotion and disease prevention in the industrialising animal production of LMICs like China and Brazil.^88^ When rising carbapenem resistance led to a resurgence of polymyxin use in human medicine around 2005, competing animal production and medical priorities meant that global regulators did not raise polymyxins’ status to that of CIAs. It was only after the discovery of the *mcr-1* gene on transferable plasmids in bacterial isolates from Chinese pigs that polymyxins were recategorised as highest priority CIAs in 2016, received specific restriction recommendations from OIE, and were banned from use as growth promoters in China and Brazil.^57 60 75^

Temporal: another challenge of prioritisation regards the inevitable temporal conflict between acute healthcare needs and the future-focused dimensions of antibiotic stewardship.^90^ There is a well-evidenced ethical dilemma between preserving drugs’ future efficacy and using antibiotics to safe-guard vulnerable populations in the present. This is highlighted by studies on the rise of multidrug-resistant pathogens in itinerant and immunocompromised HIC and MIC populations^91–94^ or of AMR proliferation as a result of high levels of antibiotic use in prolonged crisis situations like the 2009 H1N1 influenza or the current COVID-19 pandemic.^95–98^ Similar dilemmas have also become apparent in campaigns of antimicrobial mass drug administration to prevent child stunting,^99 100^ against onchocerciasis, lymphatic filariasis or malaria,^101^ in the mass administration of azithromycin against drivers of childhood mortality in Tanzania, Niger and Malawi,^102 103^ and to prevent scabies and impetigo on the Solomon Islands.^104^

Answers to this temporal dilemma vary and reflect the perceived severity of need, cultural preferences for specific forms of use, and economic considerations. In some cases, the potential longer-term risk to public health of antibiotic resistance has been favoured over immediate clinical needs.^105^ However, in the main, present needs have overridden future-focused stewardship concerns. While there is no reason not to use antibiotics to save and improve lives, they have often been used as a ‘quick fix’^106^ to symptomatically control rather than eliminate underlying drivers of infection in human, animal health and plant production systems. The tendency to see antibiotics as ‘quick fixes’ has helped drive AMR and often distracted from investment in more sustainable forms of infection prevention like effective and affordable health, Water, Sanitation and Hygiene (WASH), and Infection Prevention Control (IPC) systems (see also Inequality below).^85^ Over-reliance on antibiotics has also been exacerbated by industry marketing of, in part, inappropriate antibiotic usage and targeted campaigning to undermine usage restrictions in high-income and low-income settings.^34 77 107^

### Implementation

A third fundamental challenge for antibiotic policy-making concerns the formulation of binding international agreements that can still be implemented flexibly in different settings.^17 108^ Recent international agreements like the 2016 UN Paris Agreement on climate change remain based on the classic so-called Westphalian model of sovereign nation-states agreeing on a set of measures, which are then independently enacted within their borders without control by other actors.^7 12 109 110^ There are a number of challenges with this model: global trade flows are not easily regulated by nation-level policies; with few exceptions like the International Health Regulations on pandemics, international organisations like the WHO cannot enforce health agreements negotiated under their umbrella; the ability of governments to implement policies varies.^8 20 110 111^ Described implementation problems are exacerbated by the widespread absence of basic data and robust metrics (see above) to inform international policymaking. For example, while it may seem straightforward to define and evaluate policy success as reductions of drug usage in HICs, these metrics will likely fail in settings without reliable consumption/usage data and where informal grey market and unregulated over-the-counter sales account for a large part of the antibiotic supply.^82 112 113^

Using an integrated political, economic, sociological, technological, ecological, legislative and industry framework, reviews of national action plans proposed after the 2015 Global Action Plan on Antimicrobial Resistance have warned of continuing gaps in applying international concepts of stewardship at the national level.^114 115^ Focusing only on international and not on actual local policy alignment can foster the creation of ‘paper tiger’ initiatives, which are not enforced—as in the case of enacted but not enforced bans of over-the-counter antibiotic sales^116^—or obscure or relabel existing practices rather than reform underlying antibiotic infrastructures.^13 117 118^ Follow-up reports by WHO, UN and World Bank have proposed solutions including international investment in local IPC, antibiotic quality assurance and access schemes, AMR surveillance, vaccination, local stewardship champions and contextualised policy-making.^10 20 21 111^ In the case of animal production, experts have proposed using AMR monitoring in sentinel pathogens and technical support from HICs as tools to incentivise global reductions of antibiotic usage.^119^ Promising access to lucrative markets and using transnational integration to promote precautionary antibiotic policies is an additional tool that was used by the UK in the 1970s, Sweden in the 1990s, and is now being considered by the EU to reduce antibiotic growth promoter and prophylactic antibiotic use in non-European countries.^34 120^

However, so far, enactment of proposed measures has been fragmentary^13^ and it remains unclear how numerous national and international calls to action with complex interlayered principles of action can be translated into effective change in settings where antibiotic access is lacking and AMR is secondary (at best) to other health concerns. Looking beyond top-down nationstate alignment, by reemphasising municipal and community-based health initiatives as well as creating new metrics for antibiotic access before prioritising stewardship may be a solution. Recent social sciences research suggests the efficacy of adaptive value-driven bottom-up reforms. These reforms move from merely sanctioning inappropriate antibiotic use to identifying the sociostructural factors driving antibiotic use and devising targeted incentives for locally tailored shifts to more appropriate antibiotic use.^121–123^ However, it remains unclear whether relying on local or even regional solutions will be able to solve the global challenge posed by AMR. Calls for behavioural change, industry reform and individualised policymaking have often been ineffective.^34 77^

### Inequality

One of the most significant challenges facing international antibiotic policy and global health frameworks more widely are significant levels of inequality between and within HICs and LMICs. These inequalities reflect historically uneven social and political opportunity as well as distribution of economic resources and disease burdens. Inequality has large implications for ways priorities are made and implemented in relation to antibiotics. Recognition of these inequalities challenges forms of policymaking that focus on metrics of drug reduction alone (see Metrics and Implementation above) or prioritise protection of HIC hospital antibiotics over LMIC needs for access to antibiotics and protein production.

The unevenness of social and political opportunities affects who can make or demand policies. At the international level, the historical dominance of HICs on relevant health bodies and many funders’ and high-level meetings’ location in HICs has led to a relative absence of the voices of some of the most affected LMIC stakeholders.^124^ There is a historically evidenced danger that this dominance of HIC voices can drown out LMIC concerns and lead to narrow policies centring on HIC concerns.^125 126^ Despite best intentions, motivations for international antibiotic policy initiatives must thus be considered uneven when framed with a health security lens—who is the ‘we’ in the need to act, and who is the ‘us’ being protected^1^?

Answering these questions is important. In the case of drug development, historical priority setting has been HIC-centric due to the greater profitability of high-income markets (with a particular emphasis on the most lucrative US market) and differing regional risk priorities.^111 127^ In the case of surveillance, antibiotic resistance and usage indicators emerge unevenly from particular locations, prioritise particular security concerns and carry particular interpretations. When shuttling between different contexts in which resistance and use might have a different significance, these abstracted numeric indicators can foster a contextual disconnect among decision-makers.^128^ The described surveillance disconnect is exacerbated by the relative lack of data from LICs and rural settings (see Metrics above). The result is a vicious circle: lacking access to equipment, current standards and scholarly literature means that disease and AMR burdens cannot be measured and published,^32^ which means that there are no data with which to build local expert capacity or inform international policy, which compounds the obscuring of difference between contexts.

Over time, decontextualised international decision making can result in policies that prioritise HIC-centric stewardship interventions like targeted drug restrictions that may prove deleterious in LMICs where infection risks are markedly higher and different.^111 129^ Limited data indicate an inverse correlation between countries' gross national income and invasive infections caused by WHO top-ranked antibiotic-resistant bacteria.^71^ Where resources are stretched thinly across health systems and infrastructure, other priorities than stewardship may be more pressing for investment. For example, IPC in health facilities and availability of effective WASH systems are essential. However, in 2017, ca. 785 million people worldwide had no access to safe drinking water, two of every seven people had no access to sanitation, and 22% of LIC health facilities had no water service.^130 131^ Indeed, investment in IPC and WASH is likely to be most effective for reducing AMR in many settings as a base on which stewardship might then be built.^10 20 132^ Furthermore, in many settings the ability to provide equitable access to essential medicines of sufficient quality, including antibiotics, remains elusive.^10 112^

Similar constraints are true for animal health which is related to issues such as food safety, food security and animal welfare. Many national and international action plans invoke enhanced biosecurity facilities, vaccination or good husbandry practices as a means to avoid unnecessary non-human antibiotic use, but they rarely set these measures as (funded) priorities contrary to raising awareness, developing surveillance or promoting responsible and prudent use, or they risk assuming inappropriate livestock production and disease management approaches in settings where economic and microbiological risks are manifestly different.^133–135^ While arguments for wider systems strengthening should not undermine statutory antibiotic reform, decontextualised policies focusing only on antibiotic stewardship and surveillance risk distracting from the even more important structural absence of adequate and situationally appropriate sanitary, veterinary and healthcare systems in many parts of the world.

## Hallmarks of good antibiotic policy

**Figure 1:**
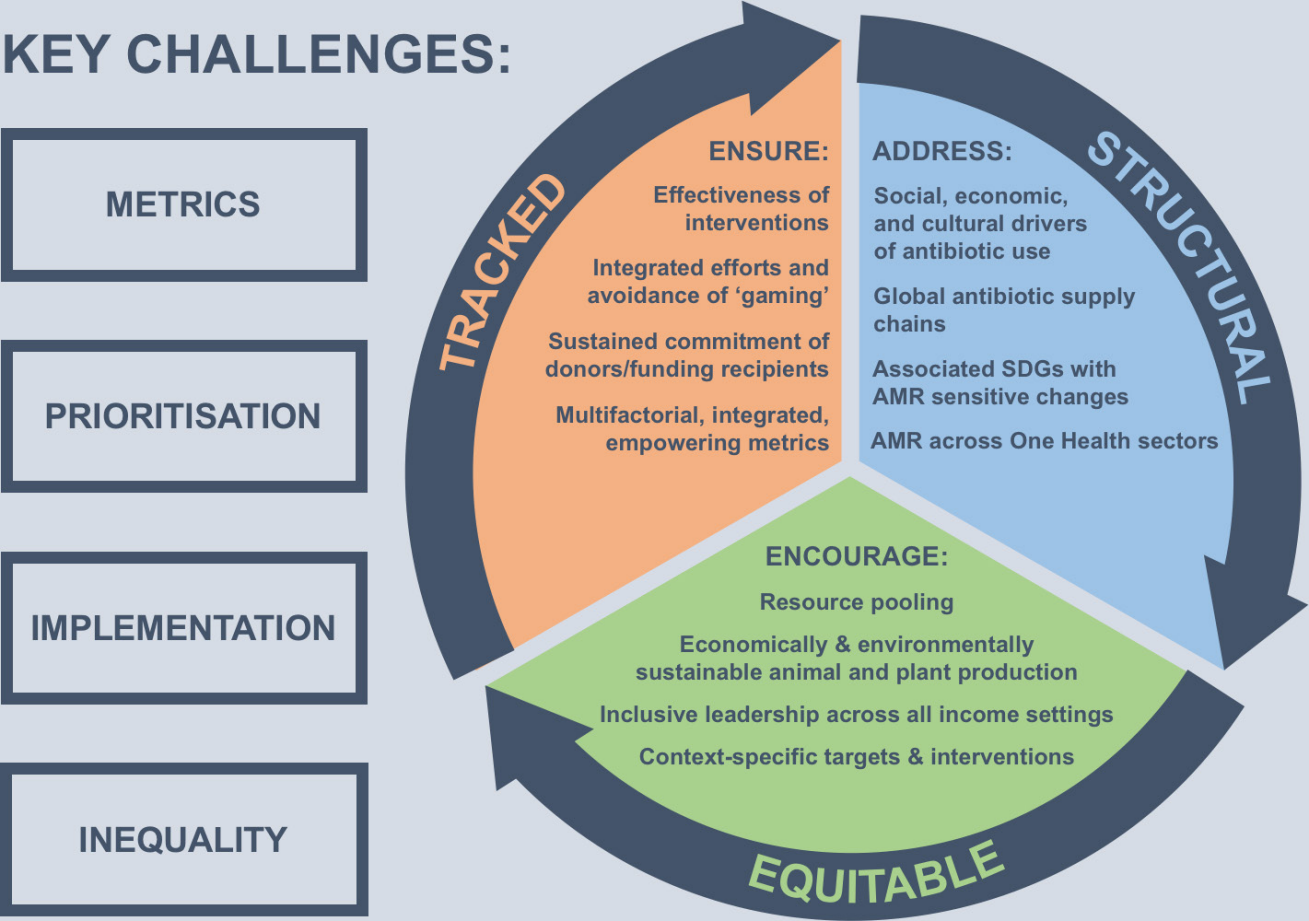
Interlinked hallmarks of successful antibiotic policy-making.

The four biosocial challenges of metrics, prioritisation, implementation and inequality require a holistic response from international antibiotic policy that is ambitious enough to improve the status quo but concrete and evocative enough to be an effective guide. Because such policies are enacted within a complex ecosystem, a broad perspective needs to inform their design and deployment to maximise effectiveness and minimise unintended consequences. It is this broad heuristic perspective, incorporating One Health, spatial, temporal and ethical dimensions that underlies the three interlinked SET hallmarks proposed here (figure 1).

- 1. Structural: International antibiotic policies should recognise and respond to the multiple aspects of global antibiotic infrastructures.^1^ Since the 1930s, antibiotics have replaced older, more expensive forms of infection control in humans, animals and plants and enabled new medical interventions like organ transplants and prosthetic joint implants. Global health and food production systems rely on the comparatively cheap ‘work’ performed by antibiotics. Reforming antibiotic use cannot be separated from broader reforms of the infrastructures that have evolved around them.
- Historically, there has been a tendency to make AMR manageable by compartmentalising problems and blaming individuals for drug overuse instead of the underlying social, political and economic factors driving antibiotic demand and dependencies (see Metrics and Prioritisation).^34 77 136 137^ In HICs, a long-standing focus on technical quick fixes and individual behavioural change meant that farmers and patients were routinely blamed for overuse without targeting wider infrastructural factors or the companies and experts supplying antibiotics.^106^ Bureaucratic divisions also meant that the same antibiotics could be subject to different regulations on farms and in clinics (see Implementation). The fact that most reforms stopped at national borders further fragmented international regulation.^76 88 136^ Since the adoption of One Health terminology by WHO and EU antibiotic regulators around 2010,^138^ nearly all international AMR initiatives have attempted to overcome described problems by integrating polices for drug regulation in human medicine, animal production and the environment. However, beyond surveillance, One Health was initially often narrowly applied to mean ‘animals’ rather than the wider environment—perhaps reflecting the absence of the UN Environmental Programme from the original Tripartite coalition of FAO, OIE and WHO. Recent European, Indian and private initiatives now explicitly target wastewaters and industrial wastes^15 134 139 140^ but implementing new standards remains challenging. Meanwhile, our scientific understanding of the relative effect of antibiotics, metals and biocides on environmental AMR burdens and of the efficacy of proposed interventions remains fragmentary.^74 141 142^
- Successful antibiotic stewardship cannot be narrow and divorced from the social and environmental contexts in which use is taking place. More effective international policy requires the abandonment of regulatory silos, as well as the adoption of context-sensitive models. It requires a broad structural approach to reforming not only the international antibiotic supply chain (drug producers) but also wider contributing global and regional consumption patterns (eg, rising global demand for protein) as well as associated socio-structural factors (eg, fractured health and WASH infrastructures, time constraints on diagnosis, profit incentives to prescribe or sell drugs) and environmental factors (eg, infection burdens, drug residues in water systems).
- As evidenced by recent successes in HIC animal production and health systems, jointly focusing on preventing disease with vaccines and improved welfare, updating the design of surveillance, hospital, animal housing systems and modifying antibiotic-seeking behaviour by patients and animal and plant producers can reduce antibiotic dependencies.^143–145^ However, high-income infrastructural starting points cannot be taken for granted elsewhere in the globe (see the Inequality section). To be effective internationally, the nature of ‘intervention’ must be less ‘AMR-specific’ and instead build up ‘AMR-sensitive’ changes^20^ which support wider UN SDGs including improved WASH, nutrition and access to affordable medical and veterinary healthcare.^10 20^ While we do not discount the importance of traditional regulatory tools like actively enforced bans of over-the-counter sales, understanding the work that antibiotics perform in non-HIC settings may also lead to an interlayering of old and new policy tools such as subsidised assurance and disease insurance schemes for farmers phasing out antibiotics, access to high-income markets for animal and plant products produced without antibiotics, public antibiotic production or certified antibiotic distribution schemes.^20 127 146 147^
- 2. Equitable: To be impactful globally, international antibiotic policies must recognise and respond to the unevenness in contributions towards and ability to tackle AMR while aiming for an equitable future for antibiotics regardless of where they are deployed. Historically, inequality and the difficulty of uniform policy implementation across HICs and LMICs have been major obstacles for international antibiotic reform (see the Inequality and Metrics section).
- Because antibiotic effectiveness is a time-limited global common pool resource and even robust national responses offer little protection from the global circulation of AMR genes and organisms, the sustained pooling of international resources is an essential prerequisite to overcome identified challenges. Similar to climate change, some countries have a greater differentiated responsibility to contribute resources to this common pool than others.^110^ For decades, populations in HICs have had greater access to the antimicrobial commons than their counterparts in LMICs. High volumes of HIC usage facilitated the historical selection for and global circulation of resistant genes and organisms while HIC companies disproportionately profited from early antibiotic sales. HICs’ historical contribution to current AMR levels and role in spreading antibiotic dependent infrastructures to other parts of the world^88 148^ entail a moral obligation to bear a higher burden when it comes to mitigating resulting problems.^110^ Similarly, recent and projected high levels of antibiotic use and production in MICs create a comparable obligation to contribute resources to mitigate problems in poorer areas of the world and for future generations.^48 119 149 150^
- Whether action is justified on the basis of historical usage, collective responsibility, obligations towards future generations, or enlightened self-interest, any international policy framework will have to include long-term financial and political HIC and MIC commitments to support antibiotic sensitive interventions in resource-poor communities. In LICs, antibiotic sensitive international support could centre on building human and infrastructural capacity by educating and employing more medical and veterinary professionals,^20^ enhancing laboratory provision and expanding access to effective, affordable, and safe vaccines, antibiotics, and WASH and health systems. From a One Health perspective, international policies should also promote economically and environmentally sustainable forms of animal and plant production as well as improve the management of waste containing antibiotics.
- Well-designed structural and equitable international antibiotic policies can generate a global win-win.^110^ Building LMIC capacity for disease control and prevention and providing equitable access to effective treatments via market reforms, subsidies or public research and development^127 151 152^ will lower both local disease burdens and rising international AMR-related healthcare costs.^10 20 111 127^ In the case of animal and plant production, equitable policies will recognise that to ensure regional food security, targets for a sustainable antibiotic-controlled production system can only come after an infrastructural groundwork is in place to reduce reliance on these substances.^119 123^
- Because of its structural dimensions, the lack of an easy target and a dysfunctional commercial research and development pipeline for new antibiotics,^127 147^ AMR has so far failed to attract the same degree of resources that organisations like the Global Vaccine Alliance (GAVI) (est. 2000) or the Global Fund (2002) have mobilised for individual diseases like HIV/AIDS, tuberculosis, malaria or polio. Achieving truly structural and equitable global AMR solutions will depend on the more effective generation and pooling of investment in safe antibiotic access, coordination of country-level policy responses and provision of effective WASH, IPC and educational resources.^127^ This role could be fulfilled by the existing Tripartite and a One Health Global Leadership Group on AMR.^153^ Another option suggested by some authors is creating a new dedicated international AMR body or pooled fund similar to GAVI.^8^ Ultimately, the structural challenges posed by AMR exceed the capabilities of any one nation. Overcoming the significant levels of inequality that have hampered previous responses will depend on intensifying international collaboration, equitably pooling resources and knowledge, and openly addressing the global disparities driving infectious disease burdens and the resulting need for antibiotics.
- 3. Tracked: Progress towards structural and equitable antibiotic policies has to be tracked to ensure ongoing effectiveness of interventions, promote integration of international efforts and motivate sustained commitment of donors and funding recipients. While a regular independent international stocktake could help ensure that policy interventions remain coordinated, equitable and up to date,^12^ the metrics informing global decision making need to be reviewed and carefully chosen.^128^ To avoid reifying existing inequalities and HIC biosecurity concerns (see the Metrics section), tracked policies should be based on and promote systems of contextual data gathering that are: multifactorial in their combination of existing and new metrics; integrated in their pooling of knowledge from different One Health and regional contexts; and empowering by conferring agency to local communities.

### Multifactorial

Achieving effective tracked interventions requires a unifying global set of multifactorial metrics. To enable the rigorous evaluation of policies, multifactorial metrics need to simultaneously take into account antibiotic access, AMR and stewardship, but avoid rewarding short-termist gaming such as relabelling disease definitions, disincentivising healthcare-seeking behaviour and decontextualising surveillance (see the Inequality section).^128 154^ Standardised terminology and transparent AMR and drug usage surveillance are essential prerequisites for the design of meaningful international interventions. However, focusing too narrowly on reducing drug usage and misuse will not curb microbial threats (see Implementation section). Vice versa, focusing only on providing drugs without supporting additional means to reduce disease burdens will achieve little in the long term. Since 2018, several ways have been proposed to integrate AMR into the UN SDG framework. The most recent proposal is to ‘reduce the percentage of bloodstream infections due to selected antimicrobial resistant organisms’ and has been recommended to go forward to the UN Statistical Commission for inclusion in SDG 3 (Good Health and Well-being).^73^ This is one promising way to address stewardship without detracting from the goals of antibiotic access and disease prevention (see the Metrics section). There is, however, an additional need for new multifactorial metrics that also encompass AMR’s environmental and animal health dimensions as well as local access to drugs, WASH, vaccines and coselection for resistance by other antimicrobial substances. The new monitoring and evaluation framework proposed by FAO, OIE and WHO in 2019 is an important step in this direction.^155^

### Integrated

In addition to drawing on multifactorial metrics, policy tracking should aim to integrate metrics across domains: taking into account the legacies of pre-existing interventions, improving knowledge integration across One Health sectors and incorporating knowledge generated in other geographical and social contexts. Enhancing the integration of international knowledge gathering will strengthen regionally nuanced decision making. Since the 1940s, successive generations of regulators have tried to manage the antibiotic commons by focusing on one form of intervention like ‘rational’ antibiotic use, reducing antibiotic use via statutory bans or non-statutory incentives and replacing antibiotics (eg, using metals in animal feeds). However, lack of cross-sectoral and international integration meant that benefits were often short-lived and had little effect on global drug usage or AMR.^34 76 137 156 157^ Regulatory silos at the national and international level have also repeatedly impeded the transmission of knowledge generated in one sector to other sectors—something that is exacerbated by the fact that animal health, medical and environmental regulators rarely interact on equal terms.^158–160^ In the case of AMR, the result was a lack of long-term strategic planning, a fragmentation of international policies and basic metrics (see the Metrics section)—and a tendency to reinvent the wheel—for example, the almost decennial recurrence of official warnings about post-antibiotic futures and ‘rational’ antibiotic use campaigns.^77 161^ Developing equitable structural policy frameworks thus not only depends on evaluating progress with multifactorial metrics but also on actively integrating knowledge throughout the One Health domains, leveraging existing national and regional policy frameworks and retaining institutional knowledge.

### Empowering

Developing multifactorial and integrated metrics will achieve little if global AMR and usage data continues to be generated and made available unevenly. Equitably boosting laboratory and data analysis capacity is a prerequisite for strengthening the ability of those on the front lines of human, animal and plant health to devise their own solutions and generate accurate data for the global community. For example, in 2010, the Danish Yellow Card Initiative required farmers and veterinarians to track and record on farm antibiotic use. The initiative complemented low-granularity sales data and helped respective communities better understand what they were doing and grow support for stewardship efforts.^54^ In LMICs, enhancing national and local capacity to generate data can have similar effects. Being able to produce and wield robust surveillance data can increase the political weight of LMIC concerns at the international level. OIE, WHO and the UK Fleming Fund are already strengthening LIC usage and AMR surveillance capacity. However, organisations still lack sufficient resources to sustainably support laboratories and countries to enable them to publish accurate country-level data.^29^ New multifactorial and integrated metrics are also needed to produce data that is meaningful in settings without formal health and veterinary care systems. In the long term, reducing reliance on decontextualised data gathering like traveller surveillance and restructuring reporting formats to better reflect varying circumstances (see the Inequality section) will significantly enhance the evidence base for truly structural and equitable international antibiotic policy-making.

## Conclusion

The past eight decades have seen antibiotic policy-makers struggle to overcome problematic metrics, narrow prioritisations, implementation deficits and global inequalities. The need for integrated architectures that act across existing silos to push evidence into action on AMR is being met through United Nations mandated mechanisms.^10^ To move beyond previous impasses, international policy will have to take seriously the infrastructural dimensions of antibiotic use, provide equitable solutions for communities across the globe and develop new forms of tracking progress that are multifactorial, integrated and empowering for the communities employing them.

**Figure 2:**
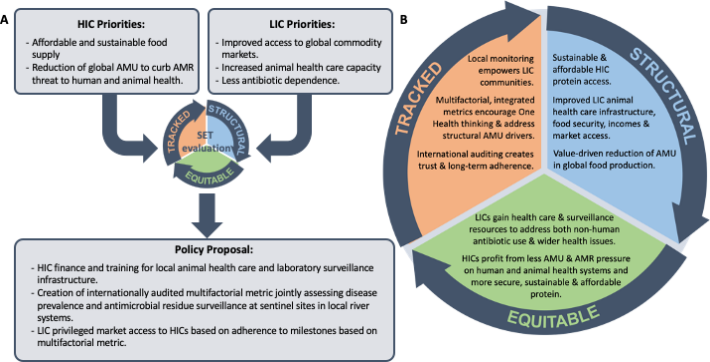
An ideal-type set of interventions for international food production. Ideal-type example of how using reflective policy-making in accordance with SET hallmarks could inform the design of equitable trade arrangements to: satisfy high income country demand for affordable sustainably produced protein; fulfil low income country calls for structural healthcare and surveillance capacity building and market access; encourage value driven. One health stewardship with multifactorial metrics integrating data on animal health with low-cost environmental sentinel monitoring for antimicrobial residues as a proxy for regional antimicrobial use (AMU). AMR, antimicrobial resistance; SET, Structural, Equitable and Tracked.

Policies that take into account antibiotics’ SET have the greatest potential to sustainably adapt the way the global antibiotic commons are managed and accessed. As highlighted in our ideal-type example of multifactorial metrics and equitable capacity building in global food production (figure 2), SET policies have the potential to foster win-win situations for HIC and LMIC participants alike.

If the ultimate goal of antibiotic policy is to reduce mortality and morbidity resulting from treatable infections, then we need to adapt our food and health systems to provide optimal access to effective antibiotic interventions when they are needed—and simultaneously reduce the need for these interventions. This dual approach requires polices that not only focus on ‘quick fixes’^106^ for existing systems and behavioural modifications at the level of the individual. Instead, policies should consistently seek to adapt the wider physical and cultural infrastructures antibiotics are embedded in.

AMR is not a problem to be solved but a phenomenon to be continuously managed. Individual policies may not address all identified problem areas or integrate each of our three intervention hallmarks. However, we believe that our multidisciplinary SET of hallmarks can serve as a compass to critically evaluate and improve antibiotic policy in the present and for decades to come.
